# Post-Graduate Education in London

**Published:** 1921-02-05

**Authors:** 


### POST-GRADUATE EDUCATION IN LONDON.

Last week it was announced that the Minister of Health, with the concurrence of the University Grants Committee, has appointed a number of eminent men to confer and report upon the needs of medical practitioners, for further education in medicine in London. It is hoped that a practicable scheme for meeting them will shortly be submitted. The Earl of Athlone will act as chairman, and the committee, among other well-known men, includes Sir Wilmot Herringham, Sir George Makins, Sir George Newman, and Sir E. Cooper Perry.

Now from a first reading of this announcement it may well be pondered as to what has become of the post-graduate schemes of which so much was heard just before, and again immediately after, the war. Have they failed? Or has the great amount of energy and individual enterprise devoted towards making London a real medical centre borne sufficient fruit to justify a national movement undeniably to establish its position?

### Praiseworthy Strides.

Undoubtedly the latter alternative must be accepted. Post-graduate teaching has made most praiseworthy strides not only towards actual efficiency, but also in provision for the graduate students' convenience. The work performed for years past by a number of London hospitals, the teaching activities of whose staffs have been largely devoted to post-graduate work, is showing a high degree of efficiency and is deservedly popular. On the other hand, the co-ordinating influence of the Fellowship of Medicine has not only helped to bring the work of the older centres into line but has allowed, also, an extension of graduate work into the wards of most of the larger London hospitals. It is a matter of history now, how, after the war, on the inspiration of Sir Arbuthnot Lane, combined with the business acumen of Sir John MacAlister, a temporary emergency scheme of teaching was inaugurated mainly for the benefit of overseas medical men visiting London on their way home. Later, with the hearty co-operation of a number of medical schools and special hospitals, the Fellowship of Medicine joined forces with the Post-Graduate Association and thus formed a permanent body to organise the potential facilities in London.

But after nearly two years' activity the general success achieved by these schemes is probably of less real importance than the experience gained in their organisation. Great improvements have been brought about, but at the same time the majority of the profession have undoubtedly realised that some form of true centralisation must be accomplished if London is, for English-speaking races, in truth to replace the Continental clinics which for decades attracted students from all over the world.

There is one factor in which the Continental centres held a decided advantage—namely, the absolute centralisation of their schemes and the comprehensive manner in which the student was provided both with exactly that instruction needed and precise directions as to where and how to obtain it. The whole thing was carried out upon a simple and efficient routine.

### The Time Factor.

The medical profession, to most of whom the time factor is of no little importance, urgently requires a similar perfection in London, and it is more than satisfactory to see that the Ministry of Health is to deal with the situation. What the ultimate recommendation of the Committee will be it is difficult to forecast, but, admitting the advances already made in the last eighteen months, it still appears that a large general hospital could, with great advantage, be devoted entirely to this phase of medical education, and act as the central pivot of the whole scheme. In their limited sphere the special post-graduate hospitals which carried their own schemes through have probably achieved better results, in quality if not in quantity, than the scattered tuition which sent a man hunting about for a lecture here and a clinic there. The University Grants Committee has distributed a million pounds in the past year and extended its aid to Metropolitan medical schools, as well as the clinical units established in certain general London hospitals. But the home practitioner needs a concrete scheme, something comparable with the academic routine of his student days.

